# Medical professionalism of foreign-born and foreign-trained physicians under close scrutiny: A qualitative study with stakeholders in Germany

**DOI:** 10.1371/journal.pone.0193010

**Published:** 2018-02-15

**Authors:** Corinna Klingler, Fatiha Ismail, Georg Marckmann, Katja Kuehlmeyer

**Affiliations:** Institute of Ethics, History and Theory of Medicine, Ludwig-Maximilians-Universität München, Munich, Germany; Rijksuniversiteit Groningen, NETHERLANDS

## Abstract

Hospitals in Germany employ increasing numbers of foreign-born and foreign-trained (FB&FT) physicians. Studies have investigated how FB&FT physicians experience their professional integration into the German healthcare system, however, the perspectives of stakeholders working with and shaping the work experiences of FB&FT physicians in German hospitals have so far been neglected. This study explores relevant stakeholders’ opinions and attitudes towards FB&FT physicians—which likely influence how these physicians settle in—and how these opinions were formed. We conducted a qualitative interview study with 25 stakeholders working in hospitals or in health policy development. The interviews were analyzed within a constructivist research paradigm using methods derived from Grounded Theory (situational analysis as well as open, axial and selective coding). We found that stakeholders tended to focus on problems in FB&FT physicians’ work performance. Participants criticized FB&FT physicians’ work for deviating from presumably shared professional standards (skill or knowledge and behavioral standards). The professional standards invoked to justify problem-focused statements comprised the definition of an ideal behavior, attitude or ability and a tolerance range that was adapted in a dynamic process. Behavior falling outside the tolerance range was criticized as unacceptable, requiring action to prevent similar deviations in the future. Furthermore, we derived three strategies (minimization, homogenization and quality management) proposed by participants to manage deviations from assumed professional standards by FB&FT physicians. We critically reflect on the social processes of evaluation and problematization and question the legitimacy of professional standards invoked. We also discuss discriminatory tendencies visible in evaluative statements of some participants as well as in some of the strategies proposed. We suggest it will be key to develop and implement better support strategies for FB&FT physicians while also addressing problematic attitudes within the receiving system to further professional integration.

## Introduction

Since the 1990s medical migration has attracted considerable interest from health and social science researchers and policy-makers alike [1]. Much of the debate has centered on motivation for migration [2, 3] and the ethical issues associated with the medical brain drain from the global South to the global West aggravating critical shortages of health professionals in many source countries [4, 5]. In contrast, the situation within destination countries and the process of professional integration of foreign-educated health professionals has long received only scant attention [6]. This has, however, changed over the last decade with the topic attracting more academic and policy attention in many traditional destination countries [7–9].

Until three years ago, research on professional integration of foreign-educated health professionals, particularly physicians who migrated to Germany, had been practically non-existent. This is surprising as in 2016, approximately 11% of all practicing physicians in Germany were foreign (41,658 in absolute numbers) [10]. While Germany’s reliance rate on foreign-trained physicians is low compared to countries like Israel (58%), New Zealand (43%) or European countries like Norway (37%), Ireland (36%) or the United Kingdom (28%), Germany is among the countries with the sharpest increases in foreign-trained doctors in the decade preceding a recent OECD migration report [11]. The number of foreign physicians in Germany was comparably low a decade ago—around 5% of practicing physicians in 2006 [12]. Restrictive policies had made it difficult to enter the German labor market even for highly skilled migrants [13]. When these policies changed, hospitals in rural, less affluent areas increasingly recruited doctors from abroad [12]. This was done to mitigate the shortage of medical doctors in German hospitals, which were facing difficulties in ensuring adequate patient care [14, 15]. Given the predicted increasing shortage of health professionals [16], this trend is unlikely to reverse. Hence, the topic of professional integration of foreign-born and foreign-trained (FB&FT) physicians is of increasing interest to stakeholders and researchers in the German context.

### Professional integration of FB&FT physicians

Integration is a notoriously vague concept. Its meaning differs according to context and user. In the context of migration, it can describe the process of being incorporated into a new system or society as well as the outcome of such a process [17]. It can be a (more) neutral, descriptive concept able to cover all kinds of ways a migrant settles into a new community, but it is more often used as a normative concept describing an ideal relationship between the migrant and the receiving society [18, 19]. Professional integration describing the incorporation of a migrant in the labor market faces similar ambiguities in its use.

We understand professional integration as a normative concept describing how incorporation in the labor market should look like (in terms of process and outcome). We follow other authors that stipulate a definition of integration that comprises the “thriving” of the migrant in the new workplace and not just becoming licensed and finding a job [7, 8]. Integration as used in the context of this study therefore implies that the migrant physician can become a fully functional and respected member of the team he or she joins. We also define integration—in contrast to assimilation—as a two-way process where both, the physician and the community he or she joins, need to make an effort and be open to change [20]. At the same time, integration has to be realized on different levels [19]: for example, policy-makers might have to adapt conditions for accessing the labor market (macro-level), hospitals might have to implement mentoring schemes (meso-level) and individual care teams have to be open to new team members (micro-level). Adequate professional integration might therefore be of importance not only to the FB&FT physician because it impacts his or her wellbeing in terms of mental health and job satisfaction [21–23]. It might also impact the wellbeing of his or her colleagues [24]. This again will likely impact retention [25] and job performance [26] of both groups. Adequate integration measures might consequently also improve the quality of patient care and patient wellbeing.

International research in this area has described various barriers for successful professional integration of FB&FT physicians. To name just the most pertinent challenges: migrant physicians working in various national contexts described educational needs regarding setting-specific competencies (e.g. language, understanding the organizational intricacies of the healthcare system and learning about cultural aspects like the focus on patient-centered care) and often complained about a lack of support structures to acquire these [27–34]. They also mentioned formal barriers in the licensure process and difficulties with being admitted to postgraduate (specialist) training programs (which in most systems constitutes the prerequisite for full licensure and unsupervised practice) [32, 35–38]. Furthermore, FB&FT physicians struggled with rejection and discrimination from patients and colleagues and further interpersonal challenges like devaluation of their internationally acquired skills [29, 35, 36, 39–41].

While most studies in this area have been conducted with FB&FT physicians, researchers have also approached colleagues, superiors and further stakeholders working with migrant physicians to describe barriers to professional integration. These studies have described similar aspects albeit with a stronger focus on limited setting-specific competencies of FB&FT physicians [6, 29, 30, 42–45]. Interestingly, to the best of our knowledge, no study has so far explored in depth the attitudes towards or reactions to FB&FT physicians of stakeholders interacting with this group. One study that touched on the subject relied on indirect assessment [46]. The authors conducted a media narrative analysis and showed the Australian physician associations to be particularly critical of FB&FT physicians. Professional associations framed employment of migrant physicians primarily as a risk to high-quality patient care. Based on this argument the professional associations wanted to restrict access to the healthcare system via increased licensure requirements. The rural community largely dependent on migrant physicians for sustaining care accused the professional associations of being motivated much more by financial interests (with them being the primary beneficiaries of sustained physician shortages) than quality considerations. Representatives of the professional associations themselves had, however, not been asked, but their attitudes were assessed via the media discourse.

This is an important gap in the literature as the attitudes and perceptions of various stakeholders in the healthcare system can themselves—as illustrated by this study—become barriers to but also facilitators of integration [47]. Skeptical attitudes might pose barriers to integration in themselves, might contribute to the lack of support structures or neglect of the issue. To foster professional integration, it will be important to understand the attitudes and perceptions of relevant actors in the receiving systems. In assessing these, this study will focus on the German context.

### Setting the scene: Immigration and integration in the German context

The importance of having robust data about physician migration for health workforce planning has long been emphasized [5, 48]. However, what is known about FB&FT physicians in Germany is rather limited. The German Medical Association does not collect data on country of birth or training, but only on nationality of registered physicians. These numbers are commonly used as approximations of the number of foreign-born and/or foreign-trained physicians [11]–which will most likely result in overestimations [12]. The most common nationalities among foreign-national physicians in Germany are European (Romania, Greece, Austria and the Russian Federation). In the last years, countries of Western Asia (most notably Syria) have gained in importance [10]. This is most likely caused by the political instability of certain areas in this region. In 2016, most foreign physicians worked in the hospital sector (81%), fewer worked in outpatient care which often means in private practice (11%) and yet fewer in other areas like medical administration (8%) [10]. The distribution differs from the general physician population where around 51% work in the hospital sector and 40% in outpatient care [10]. It has been hypothesized that the high investment costs of private practice discourage foreign doctors from working in outpatient care [12]. No recent data exist with regard to where in Germany FB&FT physicians practice. A study published in 2010 showed the growth in numbers of foreign-national physicians to be higher in Eastern Germany than in Western Germany [15]. As recruitment efforts are most pronounced in rural areas, it can be assumed that most FB&FT physicians practice or at least start practicing there [12].

Licensure is the first hurdle towards integration FB&FT physicians have to take. There are different rules for physicians who received their medical training in countries of the European Union (EU), European Economic Area (EEA) or Switzerland as compared to physicians trained in other (so called third) countries. The qualifications of physicians trained in EU/EEA countries or Switzerland will be automatically recognized when adequate command of the language can be certified. Physicians trained in other countries will have their qualifications examined for equivalency to the German medical studies [49]. The German medical curriculum requires all medical students to go through six years of theoretical and practical training (including an internship of 48 weeks in the last year) [50]. This training is supposed to convey relevant clinical, organizational, systemic, economic and ethical-legal-historical knowledge and skills [50]. In case the foreign medical training is not considered equivalent, FB&FT physicians will have to take an exam to prove equivalency of their acquired competencies [49, 51]. While waiting for equivalency to be reviewed, physicians can receive a temporary licensure of up to two years (which might be limited to one particular position). Because the licensure process is regulated by the federal states (within the limits of European and national legislation) it can differ across states (e.g. with regard to the interpretation of equivalency). This has been particularly disputed with regard to language requirements that for some states have been criticized as too soft [52]. For that reason the Conference of Health Ministers recommended in 2014 the implementation of uniform requirements, including a C1-level command (according to the European Framework of Reference for Languages) of the technical language needed in the medical context [53]. While there are still some differences between the states, most have now converged on these requirements [54]. For physicians having to take the equivalency exam (Kenntnisprüfung), there are several private initiatives offering preparatory training. These trainings are, however, not offered on a large scale and have to be paid out of pocket [55]. Some FB&FT physicians furthermore find the lengths of the licensure process particularly troublesome [56]. When licensure is completed, the regional medical associations oversee the physician’s professional conduct of medicine according to their professional codes of practice.

Barriers for professional integration have also been described for the time post-licensure once physicians enter the workplace, particularly limited setting-specific competencies and interpersonal challenges [56–59]. In this the German studies echo international findings. With regard to attitudes of co-workers, it has been shown that FB&FT doctors struggle because they face demeaning comments and rejection as foreigners, but also because they feel that their work and abilities are preconceived as deficient [56, 57]. Interestingly, in a recently conducted survey in the German state of Saxony participating foreign-national physicians expressed particularly low satisfaction regarding interpersonal aspects of their job like “work atmosphere” or “relationship with co-workers” [22]. Furthermore, support in handling these interpersonal issues, but more importantly in acquiring relevant competencies is largely not offered. A survey conducted in the German state of Bavaria showed that hospitals rarely offer support, e.g. in terms of language classes, while most agreed that there would be a need for support programs [60]. One interview study conducted with decision-makers on the hospital level in Hamburg concluded that migrants are not perceived as a special target group for personnel policies [59]. Another interview study hypothesized that hospitals in rural areas are more likely than those in urban areas to provide support measures in an attempt to retain physicians in presumably less attractive environments [58]. Either way, it can be assumed that support is largely insufficient.

While more and more is known about the professional integration of FB&FT physicians in Germany, the German like the international discourse lacks an assessment of the perspectives and positions of relevant actors of the receiving system. The studies involving stakeholders in the German healthcare system other than FB&FT physicians were primarily aimed at identifying recruitment strategies and support mechanisms implemented on the hospital level [58–60]. Although one paper touched on the perceived role of FB&FT physicians, the assessment was superficial and limited to administrative staff of hospitals in one particular city (Hamburg) [59]. The goal of our study was to better understand attitudes, perceptions and reactions of stakeholder and to explore how they form and justify their opinions about FB&FT physicians practicing in Germany. To gain these insights, we conducted a qualitative interview study with stakeholders who work with and shape FB&FT physicians’ working environment.

## Materials and methods

A qualitative research design proved to be the most adequate for our exploratory research question. We describe our methods following the consolidated criteria for reporting qualitative research (COREQ-Checklist) [61].

### Methodology

Our study is situated within a constructivist research paradigm [62] which assumes that “social reality is multiple, processual and constructed” and that “we must take the researcher’s position, privileges, perspectives, and interactions into account as an inherent part of the research reality” [63]. Our study seeks to explore how stakeholders in the healthcare system construct meaning in relation to FB&FT physicians, including their role and performance in the workplace. We utilized methods from constructivist grounded theory [63] and situational analysis [64] which allowed us to describe participants’ subjective, yet socially influenced constructions. As is typical for this type of research, we did not start from a preconceived theory, but established our theory inductively from the data.

### Methods employed in data gathering

We conducted semi-structured interviews with various stakeholders. We preferred phone interviews over face-to-face interviews. We presumed that interviews about FB&FT physicians’ work might be quite sensitive given the current German political and also historical context. We assumed that participants might be more inclined to express their opinions in the anonymous setting that of a phone conversation. Thereby, we tried to minimize social desirability bias in participants’ answers. Furthermore, phone interviews increase the chance of recruiting participants like physicians and policy-makers who have a highly demanding work schedule [65, 66].

We developed an interview guide with the help of the SPSS-method according to Helfferich [67]. Interviews started with an open question about the participants’ experiences with practicing FB&FT physicians. Specifically, we asked participants from hospitals “What experiences did you have with migrant physicians in your hospital?” or with interview partners involved in policy-making “What were your points of contact with migrant physicians?”. We then focused on problems that were experienced and/or perceived in the work of FB&FT physicians, how these problems should be resolved and who should be considered responsible for implementing change strategies. The last question was included as this study is part of a larger research project that also addresses perceptions on distribution of responsibility for implementation of integration strategies. These findings will be reported elsewhere. The interview guide was pilot tested. It was adapted to the specific context of each interview.

### Sampling & recruitment

We did not fully apply theoretical sampling in the phase of data collection, but we purposefully sampled our participants. We followed a maximum variation sampling strategy [68] in a preconceived sampling plan in order to capture a broad spectrum of perspectives. We wanted to include the perspectives of those stakeholders who participate in shaping FB&FT physicians’ work experience either directly or indirectly. Accordingly, we included people from—what we construct after Clarke [64]–different social worlds within the social arena of the healthcare system: the world of health policy-making and the world of healthcare provision in hospitals. We purposefully included FB&FT physicians in our sample. We did not include patients for ethical reasons, due to their vulnerability. We could have included former patients, but we were unable to identify and approach suitable candidates.

We chose different recruitment strategies for the different participants. Policy-makers were identified through official documents or experts and contacted directly by one of the authors. Furthermore, we included two exemplary hospitals located in rural areas in two different states of Germany (Bavaria and Rhineland-Palatinate), which employ numerous FB&FT physicians. Gatekeepers, in one case the medical director, approached participants within the hospitals. A few participants from other hospitals were approached by gatekeepers whom we knew through our professional network.

### Data collection & sample

We conducted 25 interviews between September 2014 and July 2015. The interviews lasted 15 to 62 minutes (mean: 27 min). Notes on the course of the interview, self-reflexivity and preliminary ideas for interpretation were taken after the interviews. The interviews were recorded, transcribed verbatim by a research assistant, controlled and anonymized by FL. The authors who conducted the interviews were female researchers in different career stages (FL was a medical student and MD candidate, CK was a PhD candidate in Medical Ethics, and KK was a psychologist and postdoctoral researcher in Medical Ethics). FL was trained in qualitative interviews by KK and CK; she conducted 16 interviews. KK and CK were experienced in qualitative interviews and in teaching qualitative methods and conducted eight interviews and one, respectively. None had any prior relation to the participants they interviewed.

Our sample included fourteen interviewees who work in hospitals and eleven from the policy world. Table 1 gives an overview of the sample. In accordance with our sampling plan, we approached the participants as representatives of specific roles. Some participants had multiple professional roles that formed their identity and perspective. We chose to categorize them according to their predominant role at the time of the interview. Data analysis was conducted after the data collection was completed.

**Table 1:** Description of the sample (n = 25).

Stakeholders by gender	
Female	10
Male	15
Stakeholders in the policy world by professional role (n = 11)	
Patient representatives	2
Representatives of nursing associations	1
Representatives of hospital associations	1
Representatives of physician chambers	3
Representatives of physician advocacy groups	2
Politicians (member of state parliament)	1
Employees of the federal ministry of health	1
Stakeholders in the hospital world by professional role (n = 14)	
Junior physicians • Thereof migrant physicians	5 2
Head physicians (superior senior physicians) • Thereof migrant physicians	3 1
Nursing directors	2
Medical directors	2
Hospital administration (human resources)	1
Administration of hospital group	1
Stakeholders from the hospital world by location (n = 14)	
Rural area, Bavaria (example hospital 1)	5
Rural area, Rhineland-Palatinate (example hospital 2)	5
Rural area, Bavaria	1
City area, Bavaria	2
City area, Berlin	1

### Data analysis

Three authors (CK, KK and FL) analyzed the data inductively in an iterative process using Grounded Theory strategies [63, 64]. We started the analysis with a messy situation map, using our knowledge about the discourse on FB&FT physicians (including our own study with FB&FT physicians [56]) and experiences from the interviews in order to open up the data. We defined the situation as “A FB&FT physician arrives at a German hospital to practice medicine” and wrote down all elements we considered relevant to the situation. We also explored the relationships between various elements (relational mapping) and wrote memos about our joint mappings and discussions [64]. These mapping exercises focused our subsequent analyses.

We read and coded openly the first two interviews, jointly discussed the codes and developed initial ideas for theoretical codes. CK and KK proceeded in developing the emerging model based on twelve further interviews. We alternated between coding interviews independently, joint conversations about interviews and implications for the model, writing memos (including diagramming), and reading additional literature to illuminate certain aspects of the model (open and axial coding). The sequence of the interviews in the analysis was theoretically driven. CK (re-)coded all 25 interviews based on the emerging model (selective coding), wrote memos and thereby added analytical depth to the model. We assumed theoretical saturation as the analysis of the last interviews (in the final stage) only prompted marginal adaptations of our model. Since the interviews were conducted in German, most relevant passages were translated by a native English speaker, some were translated by the first author who had studied in the UK.

### Research ethics

The Ethics Commission at the Medical Faculty of the Ludwig-Maximilians-Universität München approved this study on the condition that participant anonymity and data protection are ensured (approval number: 076–14). No full review was required. To ensure anonymity, we decided not to link interview excerpts with particular roles, but only point out whether they were shared by a representative of the policy or hospital world. Participants were informed about the purpose of the study as well as data protection and gave their informed consent. Due to the exploratory and open nature of our research, we could only provide broad information about the research questions which were refined at later research stages. However, this is common in explorative qualitative research and adequate management of these situations is still being debated [69]. All participants were asked to provide their written consent prior to the interview via e-mail, mail or fax. 21 provided written consent, while four participants consented orally.

## Results

### Problematizing FB&FT physicians’ work performance using professional standards

We reconstructed problematization to be a central feature of the discourse on FB&FT physicians’ work. Although participants tried to moderate their statements by rejecting generalizations upfront, their answers to our first open question mostly focused on problems:

*One cannot really generalize here, but it depends on the individual person. One just has the problem, well, for one thing, the language problem. That is a central problem. […] That really is the biggest problem. The second biggest problem, if we start talking about problems, is the medical qualification*…

(Representative of hospital world 1)

We were then particularly interested in understanding how participants arrive at and justify their (negative) evaluations of FB&FT physicians’ work performance. The central process that we identified during our analysis was the comparison of the physicians’ work performance with assumed professional standards (PS). With PS we mean articulations of normative requirements that *in the perspective of the respective participant* should be fulfilled by all physicians practicing in Germany. This means that not all PS referred to are indeed codified or accepted in the German context. Participants, however, assumed that these are established standards. The standards formulated are inherently linked to the professional role of physicians which is characterized by a high level of responsibility:

*Because this isn’t just like going to get the spark plugs in your car changed at the auto shop. This comes with a lot of responsibility and the fact that these colleagues just get unleashed on mankind with basically just a document in their hands, that amazes me every time*.

(Representative of hospital world 2)

PS consist of the description of an ideal (behavior, attitude or ability) and a tolerance range. While participants seem to assume that physicians should strive for the ideal, a physician’s behavior, attitudes or abilities remain acceptable as long as they lie within the tolerance range. If behavior falls outside the tolerance range, it is considered unacceptable constituting a call for action to ensure that PS are upheld in the future. If a physician does not confirm to PS long-term his or her employment contract might be terminated. In the following quote a physician evaluates her FB&FT colleagues’ work and refers to a PS requiring certain language abilities. She thereby compares the language abilities of a junior doctor born and trained in Poland and employed by the hospital to guest doctors. These so called guest doctors have often received their training in Arab countries (as specified later in the interview) and are in Germany on a medium-term scholarship from their respective countries of origin:

*So the one that I’m thinking about just now, the one from Poland, that, how should I put this, well, she isn’t even noticeable. Except for the fact that she has a bit of an accent, other than that you can’t really notice, well, I can’t really say much there. I mean she’s just like a normal colleague. The others, the guest doctors, that’s a little different, but relatively quickly/ that’s because of the language barrier. So, I mean you get the feeling, let’s put it this way, that you can’t work together with them like you would with other colleagues. […] So if you don’t know if one/if they/when you somehow split up the patients or the work, […] how they would, essentially, be able to communicate with the patient*.

(Representative of hospital world 3)

She basically argues that while it is intolerable to be unable to communicate with the patient, having an accent is not ideal, yet acceptable, even hardly noticeable. We hypothesize that the further away a behavior lies from the ideal, the more it attracts the observer’s attention.

**Fig 1:**
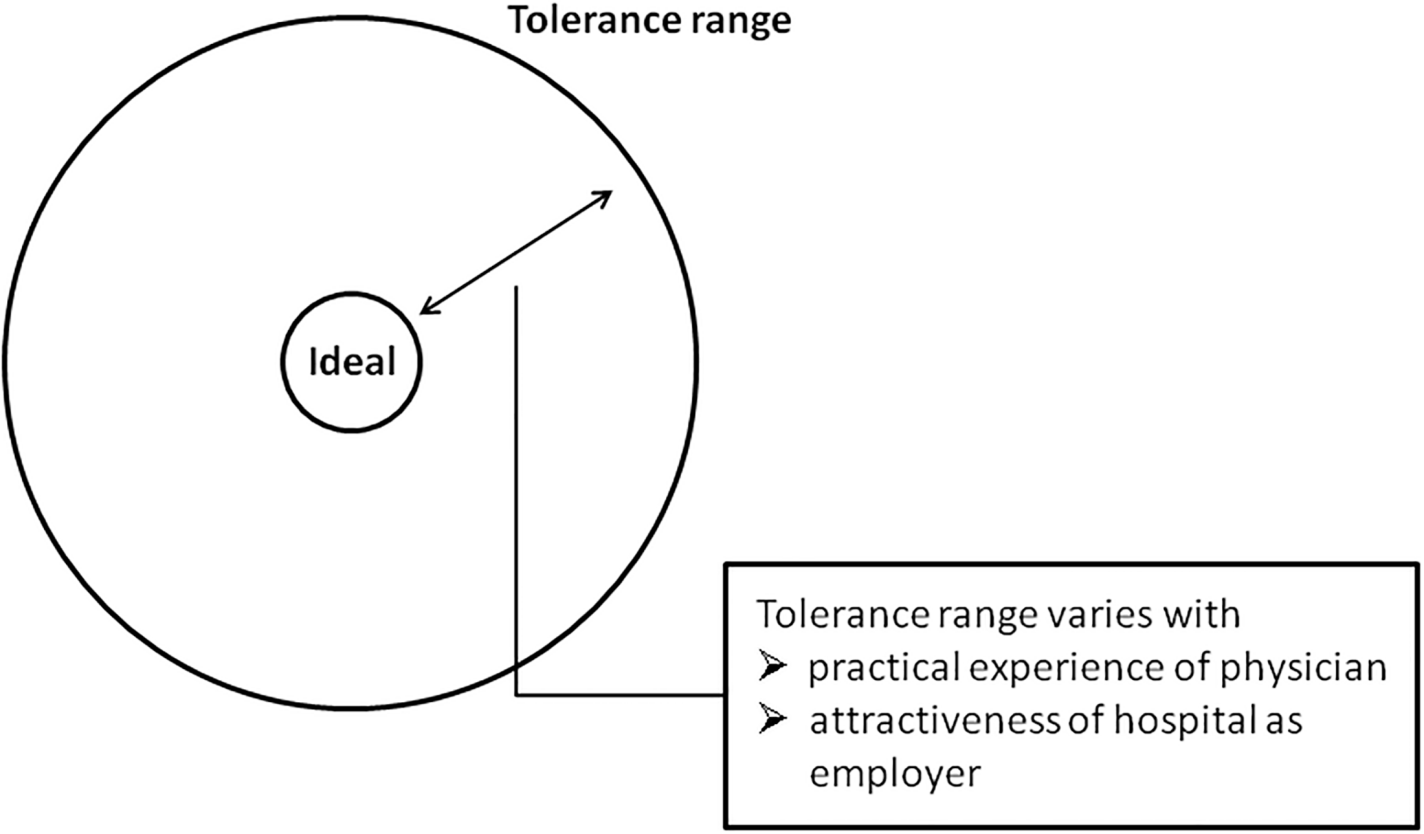
Professional standards and dynamic adjustment of tolerance range.

PS are dynamic regarding their tolerance range which can be extended under certain circumstances (see Fig 1 for visualization). Junior doctors starting medical practice, for example, are granted a wider tolerance range, which is narrowed down over time:

*He has to be empathetic. His professionality has to be coherent. This is clear, of course. But I certainly can’t expect that from a junior doctor, who is just starting out. […] And then, when a doctor doesn’t function and nevertheless, you try for a certain amount of time, but you always have the same problems with that man or woman and then you just have to draw the consequences*.

(Representative of hospital world 4)

Reasons to broaden the tolerance range can lie with the FB&FT physician, but also with the working environment. Participants described instances when they had to broaden their tolerance range because they did not have adequate applicants for a position. This difficulty was sometimes attributed to the rural location of the hospital which is often perceived as unattractive by young doctors. Another reason might be limited advance training or career opportunities:

*Some just look for opportunities, which allow them to be even lazier. And work really is delegated from A to B and from B to C and back again, and in the end nothing happens. And because we cannot, to some extent, afford to issue warnings or ultimately to terminate contracts right now, it turns into a vicious circle, where it becomes difficult to somehow manage*.

(Representative of hospital world 2)

This adaptation of the tolerance change is clearly not voluntarily chosen, but forced upon respective participants by the shortage faced in the hospital or ward.

### Invoking behavioral and skill/knowledge standards

The PS referred to by participants fall into two categories: skill/knowledge standards and behavioral standards. Skill/knowledge standards define what kind of theoretical knowledge and relevant practical abilities a physician must have acquired in order to be able to practice medicine adequately. Participants mentioned that FB&FT physicians did not always have sufficient language abilities. They also discussed limited knowledge about the organization of the healthcare system and the care processes implemented in the respective hospital. Gaps in relevant medical knowledge and practical clinical experience were also addressed. In all but one interview language standards were addressed when working with FB&FT physicians. Participants often implied that this was the biggest challenge. The following quote elaborates on the insufficient clinical skills of one exemplary group of FB&FT physicians:

*The clinical deficit, which they may also have because of their education, well, because not every degree program is structured like they are here in Germany, that clearly needs to be said. For example, they don’t do an internship in Romania […] So, it starts out highly theoretical and then they’re done and then, basically, they go out and see a patient for the very first time. We try to make up for this as best we can*.

(Representative of hospital world 5)

Participants also frequently referred to behavioral standards in criticizing FB&FT physicians. We conceptualized behavioral standards as presumably established norms regarding how a physician should act or comport him-/herself in certain circumstances in the context of the German healthcare system. The behavioral standards alluded to were quite diverse (Table 2 provides an overview). We provide three examples in which FB&FT physicians’ behavior deviated from certain behavioral standards as perceived by our participants.

**Table 2:** Professional standards invoked in evaluating FB&FT physicians.

Skill/knowledge standards	Good German language skills (written and verbal)
	Understanding of the organization of the German healthcare system and care processes in the hospital
	Adequate medical knowledge and practical clinical skills
Behavioral standards	Commitment to (advancement of own competencies for) patient care
	Equal treatment of male and female colleagues and patients
	Being responsive to patients’ (sociocultural) desires and needs
	Involving patients in clinical decision-making
	Not favoring any particular patient group
	Acting cooperatively as part of a care team (professional groups referred to were primarily other physicians and nurses)
	Not forgoing life-prolonging treatment
	Not rejecting to treat a patient for religious or moral reasons
	Not considering resource use in clinical decision making
	Being honest about (limits of) one’s own competencies
	Being independent in specialist training
	Being able to give and receive direct criticism

One participant criticized a male FB&FT colleague who was reluctant to insert a urinary catheter in a female patient. The participant presumed that religious norms regarding male-female interaction prevented the doctor from performing a necessary medical procedure:

*We really try to, well, accept how these junior doctors behave differently for religious or moral reasons. We try this but only to a certain extent. It cannot and should not be the case that a doctor does not perform any type of procedure or anything like that on the grounds of religious or moral reasons. […] It is, for example, for one of our junior doctors of Muslim origin [difficult], for example, to insert a urinary catheter*.”

(Representative of hospital world 6)

Based on the interviews alone, we were unable to determine whether this particular physician did not provide the necessary medical procedure on the grounds of his religious belief. The interpretation of the observed action as being religiously motivated could also have resulted from prejudices. Inexperience due to a lack of training in the country of origin might be an alternative explanation. In any case, in this comment a certain behavioral standard (“It is inappropriate to reject treating a patient for religious or moral reasons”) becomes manifest and is referred to in order to criticize the physician.

The following quote criticizes a group of FB&FT physicians for lacking the required commitment with regard to patient care. The participant thereby generalizes his specific experience with one or several FB&FT physicians to the abstract group of “Southerners” reinforcing a negative stereotype that is well-known in Europe:

*What is also noticeable is the resilience, in part, of Southerners. You get the feeling that there are a couple of them who only do as much as necessary. That they tend to back away or they’re not approachable or they turn their pagers off and can’t be reached*.

(Representative of hospital world 4)

Another participant expected from junior doctors a high level of independence in acquiring new knowledge during specialist training. He criticized that some FB&FT physicians did not live up to this standard.

*So for them it is simply normal that one receives, so to say, intensive teaching in hospitals. There is a sense of entitlement that could often not be fulfilled / so what I mean is that junior doctors enter [specialist training] with the same expectations as of universities. That when you start somewhere, that someone explains to you everything, so to say, from the basics to the details. And this is not possible while ambulatory services have to be upheld as well*.

(Representative of hospital world 7)

As one example illustrated particularly well, some statements attributed particular deviations from PS to a specific (e.g. religious, ethnic, national or geographic) group of FB&FT physicians—sometimes building on and reinforcing well-known stereotypes about these groups. Some participants associated limited engagement and insufficient ability to deal with direct criticism with Southern European background. Some linked being born and trained in Eastern Europe with deficiencies in clinical training and language. The group most often referred to (although still comparatively few instances were noted) were physicians from Arab countries and physicians of Muslim background. One participant discussed animosities among colleagues from different Arab countries complicating clinical teamwork. Most often participants addressed the standard of treating male and female superiors, co-workers and patients equally, as the following comment exemplifies:

*I will try to put it blatantly: There are countries in which the cultural understanding of equality of the sexes is different from ours. And this can be noticed especially with physicians from Arab countries, that we, for example, more often than normally observe problems when it comes to accepting female superiors*.

(Representative of policy world 1)

As a general worry this issue was raised in the hospital and policy world. Participants working in hospitals reported conflicts between FB&FT and female superior physicians because instructions were sometimes contested or ignored. Conflicts were also reported with the predominantly female nursing team that did not feel respected (enough) by certain physicians of said backgrounds. For one participant these observations led to a more general hesitation regarding the employment of physicians from Arab countries:

*At the moment many physicians from the Arab region start here/ I personally at the moment do not know at all how to handle/ how to judge this regarding the quality and regarding the culture or whether that will just result in more problems*.

(Representative of hospital world 5)

However, other participants explicitly refused to make generalizing statements, but pointed out the personal differences of their co-workers:

*So the motivation that the doctors who came to me had and their medical education, these were/ that was all very heterogeneous/ so you can’t tie that to a specific country, because that was very/yeah, these were very different characters*.

(Representative of hospital world 7)

### Suggested strategies for managing deviations from professional standards

Participants proposed several strategies to manage deviations from PS. We identified three types of strategies: (a) minimization, (b) homogenization, and (c) quality management (see Table 3 for overview).

**Table 3:** Strategies proposed to manage deviations from professional standards.

Minimization strategy	Change selection criteria for medical students	
	Increase medical school capacities	
	Improve working conditions in hospitals	
Homogenization strategy	Create parallel system in which physician care for patients of the same ethnicity/nationality	
Quality management strategies	Support Integration	Offer additional training opportunities outside of hospitals
		Enable hospitals to implement structured on-the-job training
		Change working procedures to allow for double-checks
	Control integration	Change licensure requirements
		Align licensure requirements across states
		Adapt licensing processes

Minimization strategies: Several participants proposed strategies to increase the number of German-trained physicians in the system. The goal of these strategies is a predominantly German-trained workforce and a minimization of FB&FT physicians in the healthcare system. This proposal rests on two assumptions: (a) German-trained physicians are less likely to exhibit behavior deviating from PS than FB&FT physicians, and (b) that increasing the number/reducing the scarcity of German-trained physicians will automatically reduce physician migration to Germany. The following quote illustrates this attitude:

*The ideal solution would be that we aren’t dependent on such problems, on this type of problem which we are discussing. So that the extent of migration could be as marginal as possible. That would actually be the desired goal. That we educate enough young professionals in our own country*.

(Representative of policy world 2)

With “problems” the participant refers to FB&FT physicians that in his perception create problems and cannot be considered an acceptable solution to physician scarcity. In order to reach the goal of increasing the number of German-trained physicians in the German healthcare system, participants suggested different strategies. They thought it would be helpful to increase medical school capacities which would presumably increase the number of practicing physicians in the long-term. They furthermore proposed to change medical schools’ selection criteria to ensure admission of students particularly interested in and apt for clinical work. The participant thereby implicitly assumed that the criteria in place at that moment were favoring students more inclined to leave clinical work at some point.

*“I think it should be made easier for high school graduates who want to study medicine to access German universities. […] I think generally that people with a second-rate Abi(tur, the high school diploma) can become pretty good physicians. Well, I mean, the bottleneck is pretty much artificially kept closed with regard to accessing medical studies. Because medical studies in Germany, they are certainly of a high quality. Accordingly, that would be the best case solution*.*”*

(Representative of hospital world 5)

Additionally, some participants highlighted the importance of improving working conditions. They assumed that this would motivate physicians to choose clinical practice in Germany over other professional activities (including practicing abroad).

Homogenization strategy: One participant suggested homogenizing doctor-patient relationships in terms of ethnicity or nationality. Basically, the participant proposed to implement parallel (or possibly better: segregated) healthcare systems for different ethnic groups or people of the same nationality—at least in large urban areas. In such a system, FB&FT physicians would exclusively provide care to patients who share their language and cultural background and accordingly refer to the same professional standards. Those are assumed to be different across ethnic/national boundaries, but the same within. The underlying assumption of this strategy is some version of cultural essentialism that attributes certain characteristics to people according to their (ethnic or national) culture. It assumes that German physicians have more characteristics in common with their German patients than physicians of other ethnic backgrounds. Or differently put: This strategy is based on the assumption that in general people within an ethnic group are more similar than people from different ethnic groups. The participant further discussed the possibility of implementing specific health insurances for different ethnic/national groups presumably meeting particular needs that arise predominantly in these groups. He also proposed to actively recruit FB&FT physicians for the nationalities/ethnicities most represented among patients in Germany:

*That also raises the question, for example, of whether to specifically look for and integrate doctors with this cultural background, in order also to take care of their people who often have language problems when dealing with our healthcare system, language and cultural problems, so to deliberately, meaning deliberately and not just to fill a gap, but rather in the sense of providing a high standard of care for citizens that have a migration background. […] Like establishing a parallel healthcare system, be it with big island practices so to say in major cities, or perhaps in some other way*.

(Representative of policy world 2)

“Island practice” is not an established expression in Germany. He later elaborates on that metaphor implying that in his vision the big “sea” of healthcare programs/institutions remains unchanged (and thereby presumably oriented towards the needs of the dominant “German” group). But there are and should be islands in that sea where care is offered for particular national/ethnic groups meeting their specific needs.

Quality management strategies: The majority of participants suggested to implement quality management strategies. Participants thereby seek to ensure that FB&FT physicians’ behavior conforms to PS either by better *controlling* or *supporting* their integration into the German healthcare system. In terms of controlling, some participants argued that the licensure procedures need to be adapted to ensure FB&FT physicians are really able to live up to professional standards. They proposed among other options to introduce stricter criteria for licensure, for example, have all FB&FT physicians retake the German final examination (Staatsexamen). Changes were also demanded for how the licensure process is handled in practice, e.g. some participants argued that it should be ensured that those assessing equivalency of qualifications are themselves qualified to do their job. Differences between states in terms of licensure criteria were also criticized and an alignment across states demanded. The worry was raised that these differences create incentives for physicians to go for loopholes in the system—states with the lowest licensure requirements—which might leave them badly prepared.

*Ideally, [licensure] should proceed in the same way Germany-wide. […] At the moment you still have small differences between the federal states, nothing big, but differences nonetheless. And there is always the worry that some kind of tourism will develop. If one realizes ‘Uff all this is, shall we say, in Rhineland-Palatinate pretty difficult, but it is a little bit easier in this other state”. That some kind of tourism will develop. And it would be desirable if nationwide [licensure] is conducted in the same way*.

(Representative of policy world 3)

Others argued for better support structures to help FB&FT physicians in acquiring relevant competencies. It was recommended to grant FB&FT physicians an adequate training before practicing medicine independently. Different possibilities to realize adequate training were discussed. It was proposed to offer (obligatory) language courses, but also courses to acquire relevant system or cultural knowledge before entering clinical practice. One participant even considered the possibility of establishing learning centers where competencies are acquired by simulating patient care:

*It would be ideal if the doctors that come here […], that they go to a, I would call it a hospital here in Germany that serves solely as a learning hospital. So, it would only have fake patients, just actors that play the role of patients and only doctors that can learn from this. They can use such a setting to become acquainted with our healthcare system and also with working in a hospital, so that when they come to the hospitals they can be put to work just like comparable, in relation to their degree, comparably in Germany educated colleagues*.

(Representative of policy world 4)

Others considered the hospital to be the adequate place for training. They argued that hospitals should implement structured orientation and/or mentoring schemes to support FB&FT physicians. Some also pointed out that it would be important to equip hospitals with the necessary financial and intellectual resources to implement such schemes. Apart from training, some participants proposed to improve working structures as to ensure that certain tasks with which FB&FT physicians are still struggling (e.g. written documentation) are controlled and corrected where necessary.

## Discussion

To our knowledge this is the first study that explores the social process of evaluating FB&FT physicians’ work performance based on interviews with stakeholders in different social worlds in the German healthcare system. When stakeholders reflected on FB&FT physicians’ work performance, we found that the latter’s professional behavior was subject to a process of close scrutiny. This entails a process of comparing their behavior to presumably shared behavioral and skill/knowledge standards and assessing the tolerability of deviation from these standards. Furthermore, we reconstructed three strategies that stakeholders considered adequate for dealing with asserted deviation from behavioral and skill/knowledge standards.

### The social process of evaluation and problematization

Studies from other countries have also asked stakeholders about barriers to the professional integration of FB&FT physicians with comparable results: limited language, system, cultural/moral and medical knowledge and clinical experience have been described as challenges when working with FB&FT physicians [6, 29, 30, 42–45]. However, these studies did not analyze the underlying processes leading to the stakeholders’ judgments which was one of the main goals of our study.

While we found actors in the care team or larger healthcare system referring to presumed professional standards in their evaluations, this might not be applicable to patients which we did not include in our sample. Studies conducted with patients show that they rely on other concepts when forming an opinion about (FB&FT) physicians: For patients, the concordance between them and their physicians—along the lines of various dimensions like social status, gender, but also ethnicity—might be especially important [70, 71]. But other factors like time taken by the physician or ways of communicating information might also impact their evaluations [70, 72].

We do not know how attitudes towards FB&FT physicians compare to attitudes towards other professional groups (e.g. engineers or IT specialists). To the best of our knowledge no similar studies have been conducted in other professional contexts in Germany that would allow such comparison. However, we would argue that perceptions and evaluations likely differ in terms of referring to a presumably shared set of professional standards. Medicine is generally assumed to be a moral enterprise because of the specific relationship of vulnerability between patient and physician [73]. The inherent morality of clinical work has made it necessary to regulate the profession with ethical codes starting from the Hippocratic Oath to the just recently revised Geneva Declaration. In this respect medicine differs fundamentally from the business or IT world. We would hypothesize that FB&FT colleagues might be particularly carefully scrutinized in morally loaded contexts such as medicine, but future (comparative) studies will be necessary to prove that point.

In the following, we analyze what the findings imply for professional integration. While it should be pointed out that many views expressed by participants were largely unproblematic, we will focus the discussion on those likely posing barriers to integration. We believe that four aspects of the interview findings warrant further reflection: (1) application of norms that are only assumed to be PS, (2) negotiability of standards, (3) prejudices and stereotypes, and (4) the adaptation of the tolerance range.

First, by making the normative grounds of judgments visible, our study permits reflecting on the legitimacy of critical remarks. Relevant for the question of legitimacy is whether the PS invoked are actually established and widely accepted in Germany. It will be particularly troublesome if FB&FT physicians are held to standards that are assumed to be professional standards, but are truly just applied—arbitrarily so—to the particular group or person. We can test the legitimacy of invoking a standard by either checking whether it is contained in legal regulation, specifically the professional code of medical conduct (strong criteria). Another indication for its legitimacy would possibly be a wide consensus regarding the standard’s adequacy among stakeholders who are affected by it (soft criteria).

In Germany, the medical profession is regulated by the Model Professional Code (and codes derived from it by regional medical associations). German-trained physicians are to be acquainted with it during their studies, although it is not specified how this training is to be implemented [74]. Most of the standards invoked by participants are indeed specified there (e.g. duty to act cooperatively, or to involve patients in decision-making) [75]. Some of the norms referred to, on the other hand, are presumably (only) tacitly accepted by stakeholders (e.g. engagement in patient care, independency in specialist training). Although careless application of tacitly accepted standards might pose integration challenges in themselves (see below), using these standards to evaluate certain situations should in most cases be considered legitimate.

Other claims are, however, clearly problematic as they invoke certain standards that are currently not widely accepted. One example would be the case where a physician is criticized for rejecting to administer a urinary catheter for religious reasons. The right to reject administering certain treatment (except in emergency situations) is explicitly granted to physicians in the Model Code [75]. It is also codified in other legal documents for morally contested measures like abortion (see §12 SchKG). Whether the right to reject treatment should be granted to doctors is ethically contested [76], however, if it is granted this rule should be applied consistently. The participant supposedly did not assume that the physician’s conviction of adequate male-female interaction provided an adequate reason for rejecting to administer certain measures. Although this position would be defendable were the physician the only one available to care for the patient, it seems arbitrary where alternatives exist. The participant’s position might therefore be interpreted as a devaluation of the physician’s divergent belief system which apparently provided less of a reason for conscientious objection than other convictions. This will likely lead to the FB&FT physician feeling less respected than his or her colleagues.

Second, when participants discussed deviations from PS the assumed direction for change was for the FB&FT physicians’ behavior to move towards identified PS. With regard to standards that are indeed codified in democratically legitimized legal documents this is arguably a valid expectation [77]. However, there are also routines that have grown as part of the ward, hospital or health system culture that could and should potentially be open to question and change as they do not touch the normative foundation of the profession. The quote demanding a high level of independence from junior doctors talks to that. In this hospital (possibly also in the whole of Germany [78]) a culture of “learning by doing” with limited instruction and supervision during specialist training has been established. Deviations from the standard of high independence or in other words a demand for instructions can highlight a different (possibly better, but more work-intensive) way of training junior doctors. Where integration is understood as a two-way process, differences in behavior should therefore not always be seen as demanding adaptation, but as a way to learn, reconsider and possibly adapt established standards and routines [20].

Third, some of our participants revealed prejudices and discriminatory attitudes. FB&FT physicians in other interview studies conducted in the German context have admitted to suffer from being mistrusted in patient care and feeling that their competencies are being preconceived as deficient [56]. Our study to an extent corroborates these findings: several participants instantly started problematizing FB&FT physicians’ work performance and thereby exhibited a largely deficiency-orientated attitude towards this group. However, we do not know whether the deficiency-orientation can be attributed to their foreignness, foreign-training or simply to them being “the new ones”. To our knowledge, no similar studies have explored perceptions of junior doctors’ work performance in general. It is therefore uncertain whether the observed evaluative processes are comparable to the processes accompanying German-trained or German-born, but foreign-trained physicians’ initial performance. Further research on stakeholder assessments of these three groups would therefore be helpful.

It is furthermore interesting that without us asking in this direction some groups of physicians were associated with certain behavior deviating from PS. Arab and Muslim doctors, for example, were criticized because they would not conform to standards of equal treatment of male and female colleagues or patients. These comments often do not just recount one experience with a particular physician, but generalize a behavior to the whole group of, for example, Arab physicians. This indicates that certain group-specific stereotypes might either influence how FB&FT physicians are seen and their behavior interpreted or which behavior will be disproportionately registered because it fits a stereotype. These kinds of generalizations—associating migrants with problems, Arab background with sexism or Southern Europeans with laziness—are problematic as they will likely impact how these groups are treated (e.g. calling in sick will more likely be interpreted as “taking a day off” for a doctor from Greece—the lazy “Southerner”–than from Germany). This was illustrated by one participant who expressed a particularly skeptical attitude towards working with physicians from Arab countries. While, for example, any physician not treating women with the required respect should be called out, stakeholders should not prejudge physicians based on described or further stereotypes. Such prejudgments can be hurtful, affect hiring practices and create conflicts among co-workers. They will thereby become barriers to professional integration. That Arab and Muslim doctors were most often singled out in problematizing statements by our participants might be an indication that these physicians experience a particularly harsh discourse. This assumption, however, has to be substantiated by further research.

Most helpful in overcoming stereotyping might be raising awareness among the receiving hospitals’ care teams. Offering team-building measures with space and support for reflecting on perceptions of and attitudes towards one another might be a good way to deal with these stereotypes. Intercultural trainings can be particularly helpful in this regard, but have so far not been taken up in any significant number in hospitals in Germany [79].

Lastly, we want to address that participants apparently felt the need to broaden their tolerance range with regard to professional standards due to the immense difficulties in finding adequate health personnel. Employing doctors who constantly do not meet their supervisors’ expectations might endanger patient safety, especially with regard to knowledge/skill standards. As our research is not equipped to estimate whether patient safety is actually at risk in certain shortage regions, further research on this topic would be highly desirable. Either way, where hospitals instead of reacting to deviations from professional standards simply broaden the tolerance range, this will also constitute a serious barrier to professional integration. Broadening the tolerance range could substitute helpful reactions such as explaining certain PS or offering support in acquiring relevant skills and knowledge. It is understandable that in regions experiencing severe shortages broadening the tolerance range can seem like the only option because physicians are already overworked. This should, however, alarm policy-makers and motivate support for respective hospitals. Unfortunately, the policy world so far seems largely unaware of or unwilling to admit existence of this phenomenon.

### Strategies to manage deviation

Participants proposed various strategies to deal with deviations from PS. Other studies had a narrower focus and examined whether stakeholders could affirm the need for better support structures and what support schemes should look like [29, 30, 43, 80–82]. Educational support programs for FB&FT physicians have also been developed, presented and critically discussed [83]. Our study, however, shows that some stakeholders—although not the majority in our sample—do not see support as the only adequate response. Instead, they wish to remove FB&FT physicians from (mainstream) healthcare altogether through ethnic homogenization of the doctor-patient relationship within the German healthcare system or by minimizing physician migration to Germany. This is again troubling from an integration perspective.

The underlying assumptions of these strategies are highly problematic. To start with the homogenization strategy: It is based on cultural essentialism which assumes certain characteristics can clearly be associated with national or ethnic backgrounds. Homogenizing patients and physicians along ethnic dimensions would accordingly reduce differences in values, beliefs and other relevant aspects, like language, in patient-physician encounters. This would presumably guarantee a high quality of the interaction. Such a strategy, however, overlooks that people’s moral beliefs, needs and even languages are not solely determined by their ethnicity/nationality. Other attributes and experiences (e.g. their migration experience, gender or age) will influence their perceptions, values and ways of communicating [84]. The diversity of values and preferences within a group sharing one characteristic (e.g. nationality) might be as large as differences between groups. Homogenization cannot guarantee that patient-physician encounters are free of conflicts or exhibit less frictions than encounters between patients and physicians of heterogeneous backgrounds. This strategy also ignores that differences in belief systems can be bridged if communication is enabled, e.g. by using language and cultural interpreters [85]. Instead of homogenizing patients and physicians along questionable dimensions like ethnicity, it seems more justifiable to foster the necessary competences and organizational structures for providing care that is responsive to patients’ individually varying needs, values and preferences—as proponents of patient-centered care have been demanding [86].

It is interesting that only one participant held such an opinion and his attitude does not reflect the (official) political discourse in Germany. The conceptual ideal advocated in recent reports commissioned by governmental institutions is “intercultural opening”. It requires institutions to become more sensitive to the possibly culturally varying needs and preferences of patients [87, 88]. Offering cultural specific, more segregated care for each national or ethnic groups is politically rather unpopular. However, the attitude might have been informed by developments on the ground: It has been shown for the Turkish community in Germany that migrants often visit doctors of their own ethnicity [89]. Furthermore, it can be observed that outpatient nursing services sometimes focus on just one ethnic patient group which is reflected in their hiring practices [90]. These choices, however, could be understood against the background of only insufficiently met needs of migrant patients in German healthcare institutions. Language and cultural interpreters, for example, are generally not available [91]. While such essentialist convictions might present a minority view, it will be necessary to raise awareness about the underlying problematic assumptions. This might be particularly important because such a strategy might seem attractive as it presumably relieves representatives of the dominant group from the need to adapt to a changing patient clientele. If these attitudes are not addressed they might hinder not only integration of FB&FT physicians, but also implementation of more culturally sensitive services.

The minimization strategy, on the other hand, is based on the assumption that training more doctors in Germany will significantly reduce the immigration of licensed doctors. Self-sufficiency, with regards to human resources for health, has long been identified by the World Health Organization as a means to curb the medical brain drain from low-income to high-income countries [5]. From a global perspective, policy-makers in high-income countries should strive to mitigate the global health worker shortage and therefore train more health professionals. It is, however, unclear to what extent training more physicians in Germany will reduce physician migration to Germany. Important push factors (e.g. political unrest, low pay) will not stop to motivate the emigration of highly skilled professionals [1]. Germany will likely remain an attractive destination country, even with increased competition on the labor market. Furthermore, opportunities for circular short-term migration for the sake of learning—as supported by the WHO [5]–might encourage migrants to apply for training positions in Germany. In addition, training more German doctors might be futile where working conditions are not addressed. High workload, relatively little pay and insufficient supervision during specialist training have motivated quite a few German-trained doctors to leave Germany and work, for example, for the UK’s National Health Service [92]. While the emigration numbers have been decreasing the last couple of years, there is still a consistent outflow of German physicians (2050 physicians emigrated in 2016, around 0.5% of all practicing physicians) [10]. Furthermore, the underlying assumption that presupposes better fulfillment of PS from German-trained physicians is in itself problematic as it rests on a negative stereotype of FB&FT physicians—as has already been discussed in detail above.

Thus, these two strategies will likely be ineffective and ethically at least questionable. They will also likely prove destructive to professional integration if pursued because proponents of these strategies prioritize forms of marginalizing or excluding FB&FT physicians over capacity building and better human resource management. It should, however, also be pointed out that in our study the majority of participants suggested quality management strategies to address deviations from PS. They proposed to better control competencies of physicians before they enter the healthcare system and/or to better support FB&FT physicians in acquiring relevant knowledge and skills. This seems to also represent the political climate: The German Medical Assembly of 2017, for example, formulated clear political demands for more educational opportunities for migrant physicians. Attendees also demanded from policy-makers a change in licensure procedures (including uniform licensure requirements across states)[93].

With regard to increasing licensure requirements there, arguably, should be a balance struck between ensuring adequate skill-level and ensuring that migrants can actually fulfil requirements. Licensure requirements can become both: a justifiable way to ensure only those with adequate competencies take care of vulnerable patient, but also a tool for exclusion. As we are not able to judge whether an adjustment of requirements is warranted, we do not know in which direction participants’ comments were pointing.

Many participants also argued that better support would be important. Support is, however, as of yet only insufficiently offered to FB&FT physicians (see Introduction) [59, 60]. At the same time, private institutions exist in Germany that have accumulated extensive experience with offering so called integration courses [55, 94]. Additionally, on the international level educational support programs have been developed. A systematic review conducted in 2015 has found 22 published interventions addressing diverse learning needs and using various conveyance strategies like presentation and discussion, mentoring, simulation and role playing as well as guided independent study activities [83]. These experiences could be built on to develop approaches for the German context to better support FB&FT physicians and thereby ensure successful professional integration. Developers of support programs should be wary that measures are not perceived as “special treatment” or reinforce problematic stereotypes. Otherwise these programs might only further separate FB&FT physicians from their care teams.

### Limitations

It is important to point out that this study with its limited sample can only describe exemplary attitudes and reactions of actors in the German healthcare system. It is therefore questionable to what extent findings are generalizable to other stakeholders in Germany. The variance in our sample was furthermore considerable. While all participants engaged in comparing FB&FT physicians to PS and almost all referred to knowledge/skill and behavioral standards, the specific (behavioral) standards invoked, but also reference to migrant sub-groups differed considerably across interviews. Attitudinal differences could in our sample not be associated with specific sub-groups of participants, but this was neither the goal of our research. Further quantitative research will be necessary to determine whether and what specific factors influence attitudes and how frequently certain perspectives are exhibited by stakeholders. Additionally, research will be helpful that analyzes more in depth possible differences between attitudes towards sub-groups of FB&FT physicians which our framing (focusing on FB&FT doctors in general) did only to a certain extent allow.

We did not use theoretical sampling in the phase of data collection, but relied on a preconceived sample plan. The preconceived plan had the strength to provide us with a broad variety of views, but some interviews did not provide us with new or deep insights in the subsequent data analysis phase. Theoretical sampling might have allowed us to arrive more quickly at our study results. Gatekeepers within participating hospitals might have been reluctant to refer us to individuals that have different opinions than they do. Participants might not have been willing to fully express their possibly discriminatory opinions due to expectations regarding social desirability, even if choosing to conduct telephone interviews has somewhat mediated this risk. On the other hand, participants might have expected us to be interested in problems because of our second focus on responsibility for implementing support structures. This might have contributed to the focus on deficiencies, although we tried to mediate this risk by starting the conversation with an open question. Constructivist studies involve a high level of interpretation and there may be other ways to read the data of this study. This being said, three researchers were involved in the analysis to ensure the intersubjective plausibility of interpretations. It would be highly desirable to employ ethnography including participatory observation, which would allow contrasting the observation of actual practice with perceptions of stakeholders.

### Self-reflexivity

Our commitment to an open society that welcomes diversity as an asset and our compassion with those FB&FT physicians who feel left alone in the early stages of their work integration might have influenced our interpretation of the interviews. In the data collection phase, the physical distance created by the phone interviews made it easier for us to manage our emotional responses and give participants space to express their opinions. During the analysis phase, we continuously reflected on each team member’s position. It helped us to fully engage with sections that we found difficult to deal with, e.g. because of their latent xenophobia. However, it still left us with a dual loyalty to FB&FT doctors and to our other participants. Our background in medical ethics might furthermore have impacted the way we focused the analysis and discussion.

In addition, we struggled with a dilemma: Although we are critical of a discourse that frames FB&FT physicians’ possibly limited setting-specific competencies as creating problems—instead of focusing on benefits, or the system as being problematic because it does not provide adequate support—we explicitly asked about problems in order to learn more about participants’ perceptions. By reproducing stakeholders’ opinions we became part of this problem-focused discourse, which we did not feel comfortable with. In current times, which the media have coined as postfactual, this paper might be mistaken as describing facts (and thereby reinforce stereotypes). Yet all it does is depicting the way certain people construct meaning.

## Conclusion

We were able to show that the stakeholders in our study put FB&FT physicians’ work performance under close scrutiny. Participants referred to professional behavioral and skills/knowledge standards to justify their critical assessments. Some participants engaged in stereotyping and held FB&FT physicians arbitrarily to only presumed “standards”. Furthermore, some participants demanded adaptation only from the FB&FT physicians and were not open to revising cultural practices and established routines themselves. They also proposed strategies to manage FB&FT physicians’ deviations from PS. By proposing minimization and homogenization strategies, some participants aimed to marginalize FB&FT physicians in the German (mainstream) healthcare system, which we argued to be ineffective, hardly justified and destructive to professional integration. We propose that deviations from PS could, however, effectively be managed by better educational programs that support FB&FT physicians in acquiring relevant competencies—as long as they are implemented in a way that does not further separate them from their care teams.

Further research should address the prevalence of and factors affecting different attitudes, but also how attitudes towards FB&FT physicians compare to German-born (and German-trained) doctors or even other professional FB&FT groups. It will be important to develop and evaluate interventions that address the care team and their possibly problematic attitudes (e.g. intercultural trainings) in addition to support programs for FB&FT physicians. Lastly, it will be essential to investigate how the physician shortage in rural areas affects the quality of healthcare in hospitals.
